# Conservative Management of Postpartum HELLP Syndrome and Intraparenchymal Liver Hematoma; A Case Report

**DOI:** 10.29252/beat-070218

**Published:** 2019-04

**Authors:** Manouchehr Ghorbanpour, Hamid Reza Makarchian, Babak Yousefi, Mehrdad Taghipour

**Affiliations:** 1 *Department of Surgery, School of Medicine, Hamedan University of Medical Sciences, Hamedan, Iran*

**Keywords:** Hematoma, HELLP syndrome, Liver, Postpartum

## Abstract

The HELLP syndrome is an important variant of pre-eclampsia which is known by triad of hemolysis (H), elevated liver enzymes (EL) and low platelet count (LP). Intraparenchymal liver hematoma is a rare and important complication of HELLP syndrome which is a life threatening condition. The incidence of intraparenchymal hematoma of the liver has been reported to vary from 1 in each 40,000 to 250,000 deliveries worldwide. Herein we report a case of intraparenchymal liver hematoma following HELLP syndrome. An 18 year- old woman with moderate to severe preeclampsia after delivery, presented with Right upper quadrant (RUQ) pain and tachycardia and significant drop in hemoglobin level. Ultrasonography revealed intraparenchymal liver hematoma. This finding was also confirmed by computerized tomography (CT)-scan. Conservative treatment was applied and the patient improved without need of any surgical intervention. Spontaneous hepatic hematoma should always be considered as a life threatening and important complication of HELLP syndrome during pregnancy and it can be managed conservatively in a hemodynamically stable patient.

## Introduction

The HELLP syndrome is an important variant of preeclampsia which is known by the triad of hemolysis (H), elevated liver enzymes (EL) and low platelet count (LP) [1]. Intraparenchymal liver hematoma is a rare and important complication of HELLP which is considered life threatening [2]. The incidence rate has been reported to vary between 1 in 40,000 to 250,000 deliveries worldwide [3]. The right liver lobe is the common site of hematoma [3] and the mortality and morbidity of both mother and neonate is increased significantly [4]. The major symptoms are sudden onset of the epigastric and right upper quadrant (RUQ) pain that usually radiate to back [4]. The situation would be diagnosed by ultrasound, computed tomography or magnetic resonance imaging [5]. The abrupt management of the condition is required to decrease the maternal and neonatal morbidity and mortality [1-3]. We herein report a case of intraparenchymal liver hematoma following HELLP syndrome.

## Case presentation

**Fig. 1:**
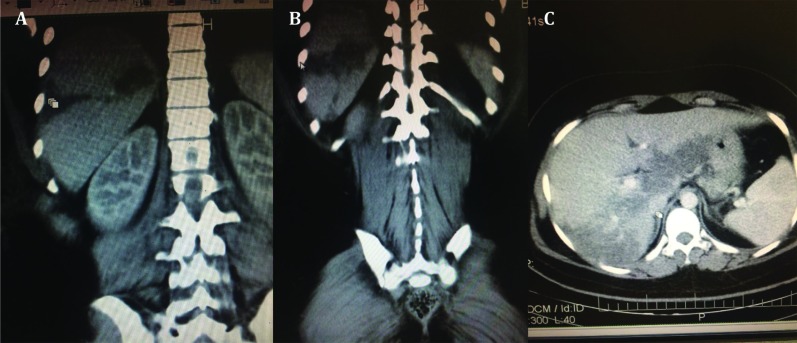
Abdominal CT-image. **A & B:** coronal view; **C:** Plain scan axial view of the lesion: demonstrating an intraparenchymal liver hematoma in the patient

A 18-year-old nulliparous woman gravida 1, para 0 with no remarkable medical history, was referred to Fatemieh Hospital Hamadan-Iran, due to labor pain and premature rupture of membrane (PROM). She had the sign of moderate to severe pre-eclampsia and delivered by normal vaginal delivery at 38 weeks of gestation. Following the delivery, she developed right upper quadrant pain, which was constant. The pain was not positional, and there was no nausea and vomiting. She had tachycardia and significant drop in hemoglobin level. Laboratory testing revealed hepatic dysfunction (AST:535 U/L; ALT:1146 U/L) and thrombocytopenia (platelet count: 77×108 per mL), with moderate to severe hemolysis (LDH:1891 U/L; HB:8/8 g/dL). She was admitted to Intensive care unit (ICU). Ultrasonography revealed intraparenchymal liver hematoma. There wasn't active bleeding and it was confirmed by computed tomography (CT) scan (Figure 1). She was transferred to surgical department and it was planned to treat conservatively. After four days, the patient improved and the pain was resolved. She was discharged and underwent follow-up monitoring. She is now alive and free from disease after a follow-up of 24 months. 

## Discussion

This case report describes a very rare case of intraparenchymal liver hematoma following HELLP syndrome. Spontaneous hepatic rupture which some time occur during pregnancy is a rare condition and can be fatal. It was first described by Abercrombie in 1844 [6]. Creating hematoma following a ruptured liver should always be considered as a life threatening and important complication of HELP syndrome [5]. The reason for this complication might be due to the mechanical trauma to the intrahepatic vasculature, and the existing hypertension seems to exacerbate this situation [7]. However, the main pathogenesis of the intrahepatic hematoma and subsequent rupture in such cases is not clearly identified. 

There is no specific and characteristic clinical presentation for the intrahepatic hematoma. The condition often presents in these patients as right upper quadrant pain [8]. Because of the rarity of this complication and its variable presentation, a misdiagnosis occurs in most cases and often accidentally discovered at imaging assessments or surgery [9]. The Tennessee Classification System outlined the diagnostic criteria for HELLP as AST (≥ 70 U/L), platelets < 100 × 109/L and hemolysis with increased LDH (> 600 U/L) [10]. Ultrasound scan is the quickest means of diagnosis, although computerized tomography is more sensitive [11]. Liver ultrasound should be performed in patients with the diagnosis of HELLP syndrome complaining of right upper quadrant, epigastric, or shoulder tip pain along with in the presence of hemodynamic instability [12]. Abdominal Magnetic resonance imaging (MRI) is a modality used in a less urgent situation or for pregnant patient [13]. 

The patients with this problems and complications must be treated in tertiary centers for the best recognition and treatment [14]. Therapeutic options in such cases depend on the severity of the problem. The choice of treatment in non-bleeding intraparenchymal hematoma with stable hemodynamic is conservative management [15]. It includes intensive care with infusion of fluid and replacing blood and its products if necessary. But in some cases, surgical interventions such as hepatectomy or even transplantation should be considered [16]. 

In conclusion, although gestational hepatic hematoma is an uncommon complication of HELLP syndrome, a high index of suspicion should be present in such patients during the treatment period . It is recommended that a multidisciplinary approach be taken to these patients. The complication can be managed without surgery in non-bleeding situations and the patients with stable hemodynamics. 

## Conflict of Interest:

 The authors declare that there are no conflicts of interest regarding the publication of this paper.
